# Successful pregnancy outcome after laparoscopic-assisted excision of a bizarre leiomyoma: a case report

**DOI:** 10.1186/1752-1947-5-344

**Published:** 2011-08-03

**Authors:** Akihiro Takeda, Sanae Imoto, Masahiko Mori, Hiromi Nakamura

**Affiliations:** 1 Department of Obstetrics & Gynecology, Gifu Prefectural Tajimi Hospital, Maebata-cho 5-161, Tajimi, Gifu, 507-8522, Japan

## Abstract

**Introduction:**

Bizarre leiomyoma is a rare leiomyoma variant that requires a precise histopathological evaluation. Especially when diagnosed in a younger woman, this tumor leads to challenging treatment issues involving fertility preservation. Owing to the low incidence of bizarre leiomyoma, there is insufficient evidence to support myomectomy alone as an appropriate management option. Also, the impact of bizarre leiomyoma on fertility is not well known.

**Case presentation:**

A 30-year-old Japanese woman who had never given birth was referred to us because of a uterine tumor with an unusual diagnostic image and was treated by a gasless laparoscopic-assisted excision with a wound retractor. Owing to an unclear margin between her uterine tumor and myometrium, a concomitant excision of adjacent myometrial tissue was required to achieve the maximum resection of her tumor. The histopathological diagnosis was bizarre leiomyoma. Seven months later, she conceived spontaneously and her pregnancy course was uneventful. At 37 weeks of gestation, an elective cesarean section was performed. Although a slight omental adhesion was noted at the postexcisional scar, her uterine wall structure was well preserved and a recurrence of bizarre leiomyoma was not noted.

**Conclusions:**

A laparoscopic-assisted excision of bizarre leiomyoma is a feasible and minimally invasive conservative measure for a woman who wishes to preserve fertility.

## Introduction

Smooth muscle tumors of the uterus encompass a variety of benign and malignant neoplasms [1]. Among uterine smooth muscle tumors, leiomyoma is the most common benign neoplasm in women of reproductive age [2]. Although most leiomyomas usually do not present a diagnostic problem, subtypes of leiomyoma mimic malignancy in one or more aspects and so are of great interest [3]. Because of the rapidly growing availability of a more conservative set of measures for women who have benign uterine pathology and want to preserve fertility, differentiating benign from malignant uterine smooth muscle tumors is becoming increasingly important when a treatment strategy is planned [1,4].

Bizarre leiomyoma, also referred to as atypical, pleomorphic, or symplastic leiomyoma, is one of a group of rare leiomyoma variants that require precise histopathological evaluation so that they are not misinterpreted as leiomyosarcomas [5,6]. Although the pathology of this morphologic variant is well established [3,5], preoperative diagnostic image findings of bizarre leiomyoma have not been described.

If fertility preservation is not required, the standard surgical intervention for bizarre leiomyoma that shows a benign clinical course is a simple hysterectomy [1,5,6]. However, owing to the low incidence of bizarre leiomyoma, there is insufficient evidence to support myomectomy alone as an appropriate management option [1,6]. Also, the impact of bizarre leiomyoma on fertility is not well known. In this report, preoperative diagnostic image characteristics and minimally invasive conservative management of bizarre leiomyoma by a laparoscopic-assisted excision that resulted in a successful pregnancy outcome in a woman who had never given birth are described.

## Case presentation

**Figure 1:**
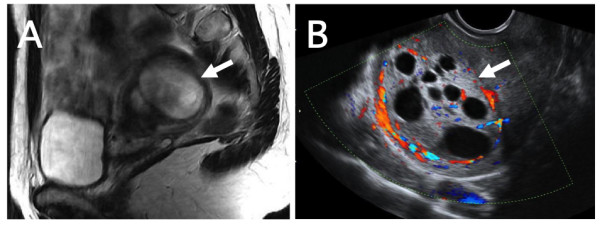
**Diagnostic image findings of bizarre leiomyoma**. (A) A sagittal T2-weighted magnetic resonance image of bizarre leiomyoma in the fundal portion of the uterine corpus. A heterogeneous ovoid tumor with an intensity signal that was mostly higher than that of the surrounding myometrium deformed the uterine cavity. (B) A transvaginal ultrasonography image of the uterine tumor with intratumoral multiple low-echoic cystic structures. Abundant blood flow around the tumor was noted.

A 30-year-old Japanese woman who had never given birth and who had no disease history consulted her physician for a check-up. Later, she was referred to our department for a uterine tumor with an unusual diagnostic image appearance. During ultrasonography, a uterine tumor that had a maximal diameter of 43 mm and that contained multiple intratumoral cystic lesions was identified (data not shown). On an image obtained by T2-weighted magnetic resonance imaging (MRI), an ovoid heterogeneous tumor in the fundal portion of her uterine corpus was noted (Figure 1A, arrow). The tumor showed a signal intensity that was higher than that of surrounding myometrial tissue, suggesting the possible presence of either tissue degeneration or hypercellularity, including malignant transformation [7]. Her uterine cavity was markedly deformed because of the uterine tumor.

We informed our patient of the potential risk of hysterectomy if a conservative excision for a uterine tumor with an unusual appearance (indicating a possible malignancy) was unsuccessful. She declined surgery at that time out of a fear of losing her fertility and chose instead to have her disease condition observed by ultrasonography at periodic check-ups. Since natural conception did not occur and the tumor gradually enlarged to a maximum diameter of 59 mm and the number of cystic structures increased six months later (Figure 1B, arrow), she hoped for a resection of the uterine tumor.

A gasless laparoscopic-assisted excision with an Alexis wound retractor (Applied Medical, Rancho Santa Margarita, CA, USA) was performed under general anesthesia in accordance with a previous description of the procedure [8,9]. The length of the suprapubic transverse incision made with the wound retractor was 2.5 cm. A hysterotomy was performed with a Harmonic scalpel (Ethicon Japan, Tokyo, Japan) after a local myometrial injection of dilute vasopressin (Pitressin; Parke-Davis, Morris Plains, NJ, USA).

**Figure 2:**
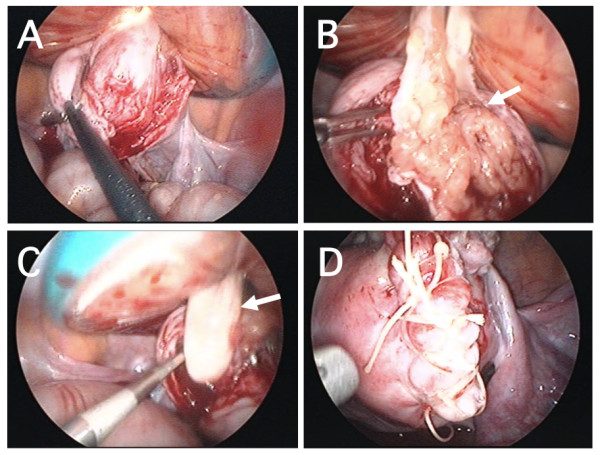
**Excision of bizarre leiomyoma by a gasless laparoscopic procedure with a wound retractor**. (A) An en bloc enucleation was difficult because of a poorly defined border between the tumor and the surrounding myometrium. (B) Prominent myxomatous change (arrow) was noted. (C) Direct palpation through the wound retractor (arrow) was useful in determining the excision line. (D) After an excision of the bizarre leiomyoma, the uterine muscular layer was reapproximated by a two-layered closure.

An en bloc enucleation was extremely difficult because of the fragile and myxomatous nature of the tumor (Figure 2A). Thus, bluntly fragmented tumor tissue was gradually excised and great care was taken not to disperse the tumor tissue in the abdominal cavity by extensive washing (Figure 2B, arrow). Owing to an unclear border between the uterine tumor and the myometrium, a concomitant excision of adjacent myometrial tissue was required to obtain maximum resection of the tumor. During this procedure, direct palpation of the tumor and the surrounding myometrium by a surgeon's index finger through the wound retractor [8] was able to reveal the excisional margin and thus minimize the damage to the normal uterine musculature (Figure 2C, arrow). Endometrial avulsion and partial defect of the endometrium occurred because of the firm attachment of the uterine tumor to the endometrium.

First, endometrial defect was closed with Vicryl Rapide sutures (Ethicon Japan). Then, the myometrial defect was reapproximated by two-layered closure with Coated Vicryl sutures (Ethicon Japan) (Figure 2D). There were no difficulties in achieving hemostasis, and the surgical procedures were completed as usual. After hemostasis was obtained, a fibrin glue-coated collagen patch (TachoComb; CSL Behring, Tokyo, Japan) was applied over the hysterotomy site through the suprapubic port to achieve further completion of hemostasis and minimize adhesion formation [8]. A J-Vac drain (Ethicon Japan) was placed in the pelvic cavity and was removed the next day after hemostasis was confirmed. Surgery lasted 82 minutes, and the loss of blood was less than 50 mL. The excised tissue weighed 20 g. The postoperative course was uneventful.

**Figure 3:**
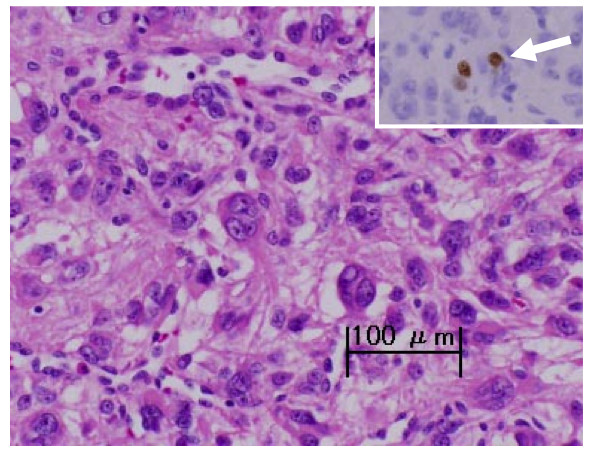
**Histopathological findings of bizarre leiomyoma**. The presence of leiomyoma cells with bizarre nuclei is shown. (Hematoxylin-eosin staining, scale bar: 100 μm.) A small number of MIB-1-positive cells (arrow) were identified (inset, immunoperoxidase staining).

A histopathological examination showed that the excised tumor was composed of numerous large atypical mononucleated or multinucleated cells with eosinophilic cytoplasm, karyorrhectic nuclei, prominent nucleoli, nuclear pseudoinclusions, and coarse chromatin (Figure 3). These bizarre cells were located in multiple foci. The stroma lying in between was focally myxoid and showed some hyalinization. Initially, leiomyosarcoma was strongly suspected. However, a low proliferative activity rate (5% to 6%) as assessed by MIB-1 (Ki-67) labeling index (Figure 3, inset) gave a final pathological diagnosis of bizarre leiomyoma with marked nuclear atypia [3].

**Figure 4:**
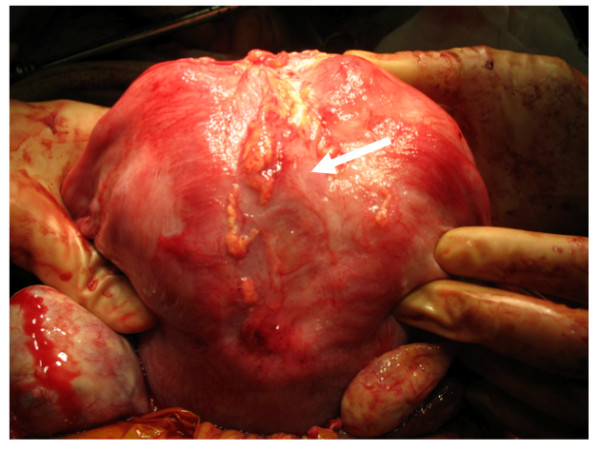
**Operative findings during a cesarean section**. A postexcisional scar at the fundal portion of the uterus at a cesarean section is shown (arrow). Although a slight omental adhesion existed, the uterine wall structure was well preserved.

After the pathological diagnosis was obtained, our patient was advised to have a regular check-up on purely empirical grounds. During ultrasonography, a remnant of uterine tumor was not evident after the operation, and the postmyomectomy scar healed well. Seven months after conservative surgery, she conceived spontaneously and underwent an uncomplicated pregnancy. At 37 weeks of gestation, an elective cesarean section was performed, and she gave birth to a healthy 3256 g girl with Apgar scores of eight and 10 at one and five minutes, respectively. During cesarean section, a slight omental adhesion to the postexcisional scar was noted. After adhesiolysis, the uterine wall structure was well preserved and a recurrence of bizarre leiomyoma was not noted (Figure 4).

## Discussion

Smooth muscle tumors are the most frequent mesenchymal tumors of the uterus [1]. Although the majority of uterine smooth muscle tumors are readily classifiable as benign or malignant (on the basis of their gross and microscopic appearances [3]), morphologic variants of leiomyoma are easily misinterpreted histologically as a malignancy [3,5,6].

Bizarre leiomyoma is one such rare leiomyoma variant that requires extensive sampling by the pathologist for a differential diagnosis from leiomyosarcoma [3], especially when the tumor is diagnosed in a younger woman, a circumstance that leads to challenging treatment issues involving fertility preservation [6]. Bizarre leiomyoma can be differentiated from leiomyosarcoma by a lack of necrotizing tumor cells and lower mitotic activity [5]. In the present case, although the initial histopathological examination suggested the malignant nature of the tumor by the presence of atypical characteristics of tumor cells, the low mitotic activity rate of the tumor cells as assessed by MIB-1 labeling index [3] gave the final pathological diagnosis of bizarre leiomyoma.

Extensive research for a pathological-radiological correlation to enhance the ability to diagnose and manage uterine smooth muscle tumors has been continued [4,7,10]. Typical appearances of uterine leiomyoma on MRI images are well established, and diagnosis is usually easy [4,10]. However, diagnostic image characteristics used to reach the precise preoperative diagnosis are unknown for such a rare but clinically problematic tumor as bizarre leiomyoma.

In the present case of bizarre leiomyoma, ultrasonography showed an unusual appearance and multiple low-echoic cystic structures within the tumor. A signal intensity that was mostly higher inside the tumor than in the surrounding myometrium on T2-weighted MRI images was another characteristic diagnostic image feature of the present case. When a surgical strategy is planned, a combination of these diagnostic image findings is the potentially important clue to narrow the preoperative diagnosis of this unusual variant of leiomyoma in a woman who wishes to preserve fertility.

If fertility preservation is not a major concern, a simple hysterectomy performed either initially or secondarily after the diagnosis of bizarre leiomyoma in a specimen obtained from myomectomy is a definitive curative treatment for bizarre leiomyoma that shows a benign clinical course [1,5,6]. However, owing to the rarity of bizarre leiomyoma, sufficient evidence to support a myomectomy for a woman who wishes to preserve fertility does not exist [1,6].

In the present case, it was assumed that bizarre leiomyoma deforming the uterine cavity was the significant cause of infertility since spontaneous conception occurred early after the operation. Although the present report suggests that conservative excision of bizarre leiomyoma could be an effective measure for a woman who wishes to preserve fertility, difficult identification of the dissecting plane because of an unclear border between the tumor and the adjacent myometrium during myomectomy should be cautioned before considering a conservative surgical intervention. Insufficient resection may cause early tumor recurrence; however, over-resection of adjacent myometrium is associated with a potentially dangerous defect of the uterine wall and may lead to uterine rupture during a subsequent pregnancy.

Reports by Sesti and colleagues [11-13] indicated that an isobaric gasless laparoscopic myomectomy using the LaparoTenser device (Lucini Surgical Concept srl, Milan, Italy) is a useful and minimally invasive measure for the management of patients who have myoma and wish to preserve fertility. In the present case of bizarre leiomyoma, a gasless laparoscopic-assisted myomectomy with a wound retractor [8,9] that was developed by us was quite useful. Even though the dissecting plane was difficult to identify because of an unclear border, the decision of appropriate dissecting margin became easy by direct palpation of the excisional area by a surgeon's index finger through the wound retractor. The reliability and safety of this procedure were confirmed by the uncomplicated pregnancy outcome in the present case.

## Conclusions

Laparoscopic-assisted excision of bizarre leiomyoma is a useful and minimally invasive conservative measure for a woman who wishes to preserve fertility, although close follow-up for an extended period of time by periodic ultrasonography for a possible recurrence is warranted. Additional conservatively managed cases with bizarre leiomyoma are required to confirm the safety and reliability of the enucleation procedure and to enable counseling of future patients who wish to preserve fertility.

## Abbreviations

MRI: magnetic resonance imaging.

## Consent

Written informed consent was obtained from the patient for publication of this case report and any accompanying images. A copy of the written consent is available for review by the Editor-in-Chief of this journal.

## Competing interests

The authors declare that they have no competing interests.

## Authors' contributions

AT collected the patient data and was involved in drafting the manuscript. SI, MM, and HN were involved in patient care. All authors read and approved the final manuscript.
